# Hyperbaric Oxygen Therapy on Long COVID Symptoms: A Breath of Fresh Air

**DOI:** 10.3390/diseases14020060

**Published:** 2026-02-07

**Authors:** Federica Zoccali, Chiara Fratini, Fiorenza Pennacchia, Francesca Cascone, Marco de Vincentiis, Carla Petrella, Christian Barbato, Antonio Minni

**Affiliations:** 1 Department of Sense Organs DOS, Sapienza University of Rome, Viale del Policlinico 155, 00161 Roma, Italy; federica.zoccali@uniroma1.it (F.Z.); chiara.fratini@uniroma1.it (C.F.); fiorenza.pennacchia@uniroma1.it (F.P.); francesca.cascone@uniroma1.it (F.C.); marco.devincentiis@uniroma1.it (M.d.V.); antonio.minni@uniroma1.it (A.M.); 2 Institute of Biochemistry and Cell Biology (IBBC), National Research Council (CNR), Sapienza University of Rome, Policlinico Umberto I, Viale del Policlinico 155, 00161 Roma, Italy; carla.petrella@cnr.it; 3 ASL Rieti-Sapienza University, Division of Otolaryngology-Head and Neck Surgery, Ospedale San Camillo de Lellis, Viale Kennedy, 02100 Rieti, Italy; 4 Interdisciplinary Department of Well-Being, Health and Environmental Sustainability (BeSSA), Sapienza University of Rome, Via delle Fontanelle, 02100 Rieti, Italy

**Keywords:** long COVID, neuro-COVID, hyperbaric, hyperbaric oxygen therapy, HBOT

## Abstract

Long COVID is defined as “the continuation or development of new symptoms 3 months after the initial SARS-CoV-2 infection, with these symptoms lasting for at least 2 months with no other explanations”, as reported by the World Health Organization. A growing number of people are dealing with a variety of lingering symptoms even after recovering from an acute infection. These can include fatigue, muscle pain, shortness of breath, headaches, cognitive issues, neurodegenerative symptoms, anxiety, depression, and a feeling of hopelessness, and therapeutic options for long COVID are investigated. The potential of hyperbaric oxygen therapy (HBOT) to improve chronic fatigue, cognitive impairments, and neurological disorders has been established; therefore, the use of HBOT to treat long COVID has also been studied. The aim of this literature search is to analyze the state of the art of a potential role of HBOT to improve chronic fatigue, cognitive impairments and neurological disorders. A literature analysis was performed, focusing on the clinical efficacy of HBOT for treating long COVID symptoms. The results from January 2021 to October 2025, using a standard registry database, showed 21 studies, including one case report, ten randomized controlled trial, eight systematic reviews and three studies regarding the molecular mechanism and markers changing after HBOT. They suggested that HBOT can improve quality of life, fatigue, cognition, neuropsychiatric symptoms and cardiopulmonary functions. HBOT is a safe treatment and has shown some benefits for long COVID symptoms. To precisely define indications, protocols, and post-treatment evaluations, we need to conduct more in-depth, large-scale studies.

## 1. Introduction

Severe Acute Respiratory Syndrome Coronavirus 2 (SARS-CoV-2) is the virus responsible for the Coronavirus Disease 2019 (COVID-19) pandemic, which has had a significant impact on health and well-being around the globe. As of March 2025, there have been over 777 million confirmed cases of COVID-19 and roughly 7 million related deaths reported worldwide [1]. In terms of clinical presentation, COVID-19 primarily shows up with respiratory symptoms, but it can also lead to a wide range of complications outside the respiratory system. Among these, acute neurological issues affecting both the central and peripheral nervous systems have been noted, including problems with smell and taste, dizziness, polyneuropathies, and, in rarer cases, encephalitis and cerebrovascular incidents [2]. Recent studies show that about one-third of patients continue to experience neurobehavioral issues within the first six months after an acute infection [3]. Additionally, a significant number of people recovering from COVID-19—whether they had mild symptoms or were asymptomatic—report ongoing issues, which are grouped together under the term post-acute sequelae of COVID-19 (PASC), often referred to as “long COVID” [4,5]. This condition is a major and persistent public health challenge, with estimates suggesting that 10–30% of non-hospitalized individuals and 50–70% of those who were hospitalized may be affected [6]. Patients who show neurological symptoms—like issues with smell and taste (anosmia and dysgeusia), cognitive difficulties often called “brain fog,” trouble sleeping, overall weakness, and psychological challenges that can diminish both physical and mental well-being—are affected by NeuroCOVID [7,8]. There is an increasing amount of research highlighting a variety of neurological and neuropsychological problems linked to SARS-CoV-2 infection, which is helping to shed light on how COVID-19 affects the nervous system [9]. One of the most reported issues after a SARS-CoV-2 infection is olfactory dysfunction, which stands out as a significant and unique symptom. In comparison, typical upper respiratory viral infections—like those caused by the influenza virus, rhinovirus, and other human coronaviruses—usually lead to conductive olfactory dysfunction. This happens due to localized inflammation and swelling in the nasal mucosa [10]. Around two-thirds of patients who experience a loss of smell more than two years after their COVID-19 show either complete or partial recovery within three years. However, the remaining one-third still struggle with ongoing symptoms [11]. The exact molecular mechanisms behind the olfactory issues linked to COVID-19 are still not completely understood. Current research indicates that this condition arises from a mix of factors, such as the virus invading the olfactory epithelium, neuroinflammation caused by cytokines that impact olfactory neurons, disruption in neurotransmitter release, and vascular damage that leads to a lack of oxygen and nutrients in the olfactory cells. Additionally, there is a persistent pro-inflammatory environment with continuous cytokine production [12]. Gaining a clear understanding of how SARS-CoV-2 infection affects the olfactory system is crucial for developing effective treatments aimed at restoring normal smell function [12,13,14]. Although most people recover fully from COVID-19, 6–12% of adults develop longer-lasting symptoms [15]. World Health Organization defined long COVID as “the continuation or development of new symptoms 3 months after the initial SARS-CoV-2 infection, with these symptoms lasting for at least 2 months with no other explanation” [16]. Patients develop ongoing persistent symptoms including dyspnea, cough, fatigue, “brain fog”, cognitive dysfunction, anxiety, depression, sleep disturbances, palpitations, postural tachycardia syndrome (POTS), and rashes that continue for more than 12 weeks not explained by another alternative diagnosis [16]. Long COVID syndrome is well described worldwide with symptoms affecting quality of life and work–life. The exact causes of long COVID are debated and it is likely that multiple mechanisms are involved [17]. Moreover, factors like viral persistence, hypercoagulability, immune system issues, autoimmunity, and hyperinflammation have all been proposed; there are currently no effective treatment protocols that address the underlying problems associated with long COVID [16]. Available therapeutic choices for long COVID are largely insufficient, and a multidisciplinary assessment is often required in the management of those symptoms. COVID-19 alters the oxidative phosphorylation process in mitochondria, which may contribute to the onset of chronic neurological disorders associated with the infection [18]. Recent studies also reveal that nearly a third of COVID-19 patients develop long-term neurological problems, highlighting the crucial importance of studying mitochondrial mechanisms to mitigate the long-term neurological effects of COVID-19 [18]. Higher antioxidant activity has been observed to be associated with greater protection against COVID-19 infection. Compounds with antioxidant properties, which support mitochondrial balance, have been shown to improve the condition of COVID-19 patients [18]. Mitochondrial biological markers, including the amount of mtDNA, electron transport chain enzyme complexes, lactate and CoQ10 levels, and indicators of oxidative stress, are promising as indicators of disease severity and prognosis in COVID-19 patients who experience neurological problems [19]. Aging further exacerbates mitochondrial dysfunction, increasing susceptibility to severe forms of COVID-19 and associated neurological complications [20]. Therefore, addressing mitochondrial dysfunction represents a potential therapeutic approach to managing COVID-19-associated neurological symptoms [20]. Recent studies have shown that patients undergoing HBOT experienced notable improvements in various areas, including overall cognitive function, fatigue levels, attention span, executive function, energy, sleep quality, psychiatric symptoms, cardiopulmonary health, endurance, and pain management. HBOT is a non-invasive treatment that involves breathing 100% oxygen in a pressurized chamber. As the pressure rises, more oxygen gets dissolved into the blood plasma, which boosts the oxygen supply to the tissues [21]. HBOT demonstrated its benefits in neurological disorders and other syndromes characterized by chronic fatigue too [21]. HBOT has the potential to lower oxidative stress and chronic inflammation, enhance endothelial function, and ease long COVID symptoms. By boosting the amount of oxygen that gets dissolved in body tissues, the combined effects of hyperoxia and hyperbaric pressure can activate genes that respond to oxygen and pressure. This, in turn, sparks regenerative processes like the proliferation and mobilization of stem cells, along with the release of anti-apoptotic and anti-inflammatory factors, angiogenesis, and neurogenesis [22]. HBOT can improve cerebral blood flow, and it can induce neuroplasticity, improve cognitive function and reduce symptoms of long COVID [22]. All of this explains why the use of HBOT can provide significant benefits and could become a clinical treatment for those long COVID conditions that do not respond to other medical symptomatic therapies, with a significant improvement in global cognitive function, fatigue, attention, executive function, energy, sleep, psychiatric symptoms, cardiopulmonary function, endurance and pain. Here we aim to better investigate the state of the art and the efficacy and safety of HBOT for long COVID symptoms.

## 2. Materials and Methods

A scoping review was conducted following the Preferred Reporting Items for Systematic Reviews and Meta-Analyses (PRISMA) 2020 guidelines. A literature search was performed using MEDLINE, EMBASE, PUBMED and Scopus Databases. The search strategy was conducted using the following terms: long COVID AND hyperbaric oxygen therapy OR HBOT. Study selection: the results were extrapolated from January 2021 and October 2025. All the clinical and case reports included an abstract available in English language, description of referred symptoms, treatment performed, outcome and follow-up on individual patients. We excluded articles with lacking information. This review included randomized controlled trials, observational studies, case reports and systematic reviews enrolling human participants with long COVID characterized by fatigue, myalgia, dyspnea, headache, cognitive impairment, neurodegenerative symptoms, anxiety, depression, and sense of despair, which were treated with HBOT. This review is limited to human studies and research papers written in English. Abstracts, reviews, editorials, and opinion articles without data were excluded. Title and abstract were watchfully examined by two authors (F.Z. and C.F.) independently, and disagreements were resolved by a discussion with a third author (F.P.). Full text of the included studies was reviewed, and data extraction was performed using a standard registry database. Epidemiologic and clinicopathologic data, registered in each study, included age, sex, clinical symptoms, treatment performed, outcome and follow-up. Studies were considered for inclusion if they included adults (aged 18 years and over) who suffered from the continuation or development of new symptoms three months after the initial SARS-CoV-2 infection. We included studies that investigated how hyperbaric oxygen therapy (HBOT) affects long COVID in our analysis. However, we decided to leave out any studies that met any of these criteria: (1) those not focused on long COVID; (2) those missing important safety and efficacy information about HBOT; (3) abstracts, editorials, or letters; (4) duplicate studies.

## 3. Results

**Figure 1:**
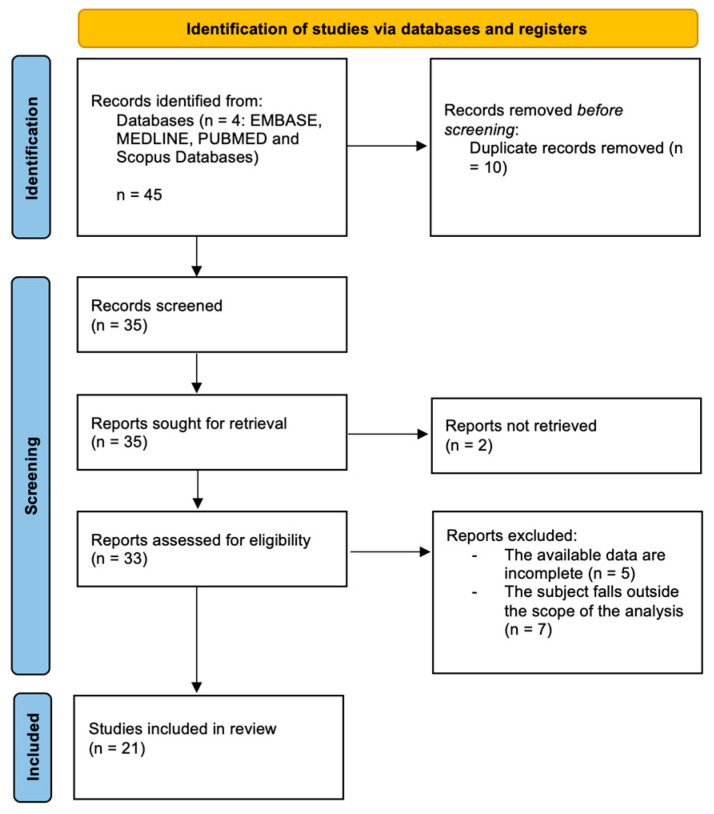
PRISMA 2020 flow-chart.

The search algorithm and review results, following PRISMA 2020 guidelines, are outlined in Figure 1. The initial search found 45 studies on the MEDLINE, EMBASE, PUBMED and Scopus databases. The removal of duplicates identifies 35 publications. All 35 papers were screened in title and abstract, and 33 manuscripts were reviewed in full text. Of these, 21 studies meet the inclusion criteria, while the remaining 12 studies were excluded. The included studies are heterogeneous, so a formal meta-analysis could not be appropriately performed. The data collected from each study were transcribed in a tabular form. Of the 21 studies selected, one was a case report [23], 10 were randomized controlled trials (Table 1), seven were systematic reviews (Table 2) and three studies regarding the molecular mechanism and markers changing after HBOT (Table 3). Several differences can be identified among the studies: some report positive results, others do not. A general summary can be reported as follows.

### 3.1. HBOT Protocol

There were significant differences in HBOT treatment protocols among the several investigations included (see Table 1). The methods of HBOT therapy vary throughout the studies (see Table 1 and Table 2). The length of each session, the total number of sessions, and the timing of the therapy following a COVID-19 infection all depend on the pressure that is applied. The studies that used pressures usually more than 2.0 ATA and longer treatment plan had better results. On the other hand, the studies that used lower pressures or did not treat people for as long did not produce promising findings. The interval between contracting COVID-19 and initiating HBOT varies across studies, making it difficult to compare the effects of HBOT. In conclusion, since a universal protocol for the management of patients with post-COVID-19 sequelae using HBOT has not yet established, it is difficult to achieve an effective comparison among the different populations analyzed in the studies reported in our literature search.

Characteristics of Patients: The recruited patients in different studies were all quite different. Some studies included adult patients, while others included younger patients. In addition, the number of males and females in each trial varied. COVID-19 symptoms patients experienced when they first became ill also differed among studies. Some patients referred severe symptoms, others milder symptoms. They also had other co-morbidities. The main problems that patients had after they got COVID-19 were different in each study. Several studies focused on issues with thinking and feeling exhausted. Studies that looked at patients who still had problems with not getting oxygen to their tissues or problems with small blood vessels often had better results with HBOT COVID-19 treatment. COVID-19 patients who experienced these issues were more likely to respond to therapy. Some studies looked at people of certain ages or genders but this was not done very often. The sample sizes for these studies by age or gender were just too small to make strong comparisons between subgroups, like age or gender. (see Table 1 and Table 2). Across the studies included in this review, individual patient clinical characteristics were frequently unreported, with most articles providing only aggregate descriptions of symptoms that improved or not following HBOT. This heterogeneity in reporting, together with the lack of standardized outcome measures and treatment protocols, substantially limits comparability across studies and increases the risk of bias. Consequently, while symptom-specific treatment effects may be inferred, universally generalizable conclusion cannot be drawn.

### 3.2. Outcome Results

The reported results were influenced by differences in measurements taken in different studies. Consistent changes in results can be observed based on subjects’ self-reported information, such as their reasoning ability, level of fatigue, and level of well-being. Physical parameters, such as lung function tests and imaging studies, do not show significant changes after the use of HBOT. The fact that the trials [29,35] are still ongoing and did not all follow up with the people over time could have also made it harder to figure out what the results really meant and to compare them to each other. Here we are looking at the effects of HBOT, and the results of HBOT can be different as we can see in the conclusions reported among Table 1 and Table 2. Hadanny et al. [24], van Berkel et al. [25], Robbins et al. [27], Zant et al. [28], Leitman et al. [30], Gonevski et al. [32] and Lindenmann et al. [33], Mrakic-Sposta et al. [39], Pan et al. [40], and Jermakow et al. [41] all reported positive outcomes following HBOT in the patient cohorts, with statistically significant improvements observed in quality of life, cognitive abilities, attention, left ventricular systolic function, and physical fatigue. These outcomes pertain to symptoms that had worsened following COVID-19. Conversely, Kjellberg et al. [26] described high frequency of adverse events following HBOT and D’Hoore et al. [31] reported no significant differences in subjective symptoms, functional scores, and cognitive performance between any groups analyzed.

### 3.3. Design of the Study

One more cause of variability in stated results found was study design. Uncontrolled tests and observational studies reported favorable impacts of HBOT more often than did randomized controlled trials, especially those using placebo treatments. Inconsistencies across the literature were exacerbated by sample size constraints and methodical heterogeneity as well. Future research should prioritize well-designed, standardized studies with larger sample sizes and clearly defined clinical and methodological parameters to adequately assess the efficacy of structured HBOT treatment protocols.

## 4. Discussion

Research on long COVID has had a slow and challenging start, and there is still not a full understanding of its complex biological processes. One proposed explanation involves immune system issues, such as abnormal T-cell activity, changes in gut bacteria, autoimmune reactions, problems with blood vessel function, issues with mitochondrial health, lingering virus, or problems with brain signaling [25]. All these changes may lead to the development of highly heterogeneous symptoms, which nonetheless share a common denominator: the underlying COVID-19 infection.

### 4.1. Neurological Symptoms

A potential explanation of the persistence of neurological symptoms, for example, might be an association with dysautonomia or autonomic dysfunction, caused by a malfunction of the autonomic nervous system (ANS) [42]. Dysautonomia contributes to cerebral hypoperfusion that leads to an overactive sympathetic system and concomitant reduced parasympathetic activity [43,44].

Because of the lack of clear understanding, treatment options for long COVID are limited and no standard therapy has been established. These patients could benefit from vagal nerve stimulation and oxygen treatment [45]. In fact, there is a growing need to find more effective treatments for this condition. Common approaches include ways to manage symptoms, rest, and coping strategies [41].

### 4.2. Actual Application and Potential Role of HBOT

In the search for an effective treatment, hyperbaric oxygen therapy (HBOT) has emerged as a promising option due to its potential to address the effects of COVID-19. Several studies have shown impressive improvements in various areas, such as quality of life, physical health, memory, concentration, and heart and lung function (see Table 1, Table 2 and Table 3). Many studies have highlighted the effectiveness of hyperbaric oxygen therapy (HBOT) in helping post-COVID-19 patients with cognitive issues, likely by improving brain plasticity and reducing brain inflammation. HBOT has been used in clinical settings since 1957 for conditions like heart surgery, decompression sickness, carbon monoxide poisoning, air embolism, and infections that thrive without oxygen. This therapy involves breathing in nearly 100% oxygen inside a pressurized chamber, which is more than two times the normal atmospheric pressure. This allows oxygen to dissolve in the blood at levels about 11–14 times higher than under normal conditions. The shift between high oxygen and normal oxygen levels helps the body improve oxygen delivery to areas with low oxygen, supports tissue repair and growth, and helps control inflammation. Currently, HBOT is recognized for treating conditions like diabetic foot ulcers, radiation injury, and decompression sickness. On a molecular level, HBOT increases oxygen levels in tissues and activates genes involved in regenerative processes, such as stem cell growth, anti-inflammatory pathways, new blood vessel formation, and better mitochondrial function [46]. Moreover, HBOT can affect blood flow to the brain and its structure, which supports brain plasticity and cognitive abilities [47]. Randomized controlled trials have further confirmed the therapeutic benefits of HBOT for post-COVID-19 patients, helping to relieve a range of ongoing physical, cognitive, and psychological symptoms, including issues with memory, heart function, and changes in brain connections [48].

### 4.3. Molecular Mechanisms of COVID-19 Acute Phase

Many of the studies we looked at suggest that hyperbaric oxygen therapy (HBOT) can be very helpful in managing long COVID symptoms. To better understand this, we need to consider the acute phase of COVID-19, marked by widespread inflammation, increased clotting, and blockages in both central and peripheral blood vessels (see Table 3). This widespread inflammatory response, often called a cytokine storm, is characterized by high levels of IL-1, IL-6, and TNF-α. These factors can lead to small areas of tissue death and inflammation in the brain, causing localized oxygen shortages [35].

### 4.4. What About Long COVID?

Long COVID, when symptoms last more than three months after a probable or confirmed SARS-CoV-2 infection, often includes issues like memory problems, fatigue after exertion, sleep issues, loss of taste and smell, and other systemic challenges. Data shows that out of 80 million people infected between 2020 and 2023, about 54% reported ongoing fatigue, often along with physical pain, cognitive struggles, or breathing issues three months after their infection [35]. Among Italian patients, 53% reported fatigue and 22% reported chest pain after two months; in a British group of 100 survivors, more than two-thirds had persistent fatigue after 4–8 weeks [35]. Like chronic fatigue syndrome, pro-inflammatory components through cytokines like TNF-α and IL-7 are also thought to impair the normal function of the central nervous system in post-COVID syndrome, leading to the various symptoms mentioned [35]. Since HBOT is increasingly used in several neurological diseases and syndromes with chronic fatigue or cognitive issues, it would make sense to apply this therapy to post and long COVID patients [35]. In fact, most studies found that HBOT can improve quality of life, fatigue, cognition, mental health symptoms, and heart and lung function.

### 4.5. What Could Be Future Directions in Therapy?

Even though the cause of long COVID is largely unknown, viral persistence, increased clotting, immune dysfunction, autoimmune reactions, and excessive inflammation have been proposed as possible causes [49]. Considering our current understanding of long COVID symptoms, HBOT has emerged as a potential treatment option [50]. The overall hypothesis about how HBOT helps long COVID is that it can reduce oxidative stress and long-term inflammation, improve issues with blood vessel function, and thus alleviate symptoms of long COVID. HBOT increases the amount of oxygen dissolved in body tissues; the combined effect of high oxygen and high pressure can trigger genes that respond to oxygen and pressure, leading to regenerative processes like stem-cell growth and anti-inflammatory and anti-apoptotic factors, new blood vessel growth, and brain cell growth [51]. HBOT can improve blood flow to the brain’s affected areas and maintain the integrity of brain structures, therefore supporting brain plasticity and improving cognitive function. Additionally, HBOT has positive effects on mitochondrial function, a key part of proper muscle function. It can also increase the number of satellite cells and regenerate muscle fibers, which promotes muscle strength. In previously published studies reviewed in this paper, HBOT has been shown to reduce inflammatory responses and cytokine levels, decrease tissue damage by lowering oxidative stress and reactive oxygen species, improve immune system function, and work together with antibiotics to have a synergistic effect against infections. It can also reduce platelet activation and aggregation in the lungs and improve lung tissue microcirculation. Although HBOT has shown several benefits for prolonged COVID symptoms, it is still difficult to debate whether HBOT treatment for prolonged COVID is effective or not. It has been shown to be effective in reducing or modifying many multi-organ symptoms in a lasting way and without side effects, but more rigorous large-scale randomized clinical trials are needed to establish guidelines, treatment protocols, and post-treatment assessments. We believe that the difficulty in diagnosing the syndrome and the lack of access to many healthcare facilities that offer hyperbaric chambers as a therapeutic option prevent clinicians from providing specific recommendations. [52].

## Figures and Tables

**Table 1:** Randomized controlled trial evaluated in the text.

Author	Year	Study Design	Patient Number	Synopsis
Hadanny et al. [24]	2024	Randomized controlled trial	31	HBOT can improve quality of life, sleep, psychiatric and pain symptoms. The clinical improvements were reported even 1 year after the last HBOT session.
van Berkel et al. [25]	2025	Prospective registry	232	The main improvements were seen in cognitive tasks. It seems that HBOT could have a beneficial impact on long COVID symptoms, but it is important to stay vigilant for any signs of the condition worsening.
Kjellberg et al. [26]	2023	Randomized controlled trial	20	High frequency of adverse events was observed but the data safety monitory board assessed HBOT to have a safety profile.
Robbins et al. [27]	2021	Case series	10	HBOT showed a statistically significant improvement in several areas, including the Chalder fatigue scale, global cognition, executive function, attention, information processing, and verbal function.
Zant et al. [28]	2022	Case series	6	HBOT made a real difference by boosting symptom scores, shortening the duration of symptoms, and enhancing overall quality of life.
Kjellberg et al. [29]	2022	Randomized, placebo-controlled, double-blind, phase II clinical trial	80	Main efficacy and safety endpoints will be evaluated at three months but the trial will continue for one year after inclusion or until withdrawal. There will also be a 4-year post-trial follow-up of health-economy.
Leitman et al. [30]	2023	Randomized controlled trial	60	HBOT promotes left ventricular systolic function recovery in PASC patients.
D’hoore et al. [31]	2025	Randomized, placebo-controlled, prospective, double-blind trial	101	After treatment there were no significant differences in subjective symptoms, functional scores, and cognitive performance between any groups.
Gonevski [32]	2024	Case series	63	HBOT delivered positive results in all observed long COVID related symptoms, particularly those associated with the nervous system, cognitive function, psychological well-being, and physical fatigue. Benefits achieved were persistent after three months.
Lindenmann et al. [33]	2023	Clinical Pilot Study	70	Physical and emotional role improved significantly, suggesting HBOT as a promising supportive tool for the treatment of long COVID.

**Table 2:** Systematic reviews evaluated in the manuscript.

Author	Year	Study Design	Studies Analyzed	Synopsis
Wu et al. [22]	2024	Systematic review	10	HBOT can improve quality of life, fatigue, cognition, neuropsychiatric symptoms, and cardiopulmonary function.
Gorenshtein et al. [34]	2024	Systematic review	3	Improvements in cognitive assessment tests and brain perfusion.
Li et al. [35]	2024	Systematic review and meta-analyses	Not indicated	The review is still ongoing (End date: 30 September 2026)
Katz et al. [21]	2024	Clinical review	8	HBOT is a safe treatment and may provide benefit.
Pawlik et al. [36]	2024	Critical review	1	A recent randomized controlled trial suggests that hyperbaric oxygen therapy (HBOT) can lead to long-lasting improvements in cognitive function, brain network regeneration, and heart health. It appears that HBOT could have both theoretical and practical implications for addressing the current physiological challenges associated with post-COVID conditions.
Joli et al. [37]	2022	Systematic review	20	Patients noticed improvement in fatigue after HBOT and enhanced external counter pulsation.
Zamora et al. [38]	2025	Systematic review	7	HBOT might be a potential option and safe treatment in long COVID syndrome patients.

**Table 3:** Studies regarding the molecular mechanism and markers changing after HBOT.

Author	Year	Study Design	Patient Number	Synopsis
Mrakic-Sposta et al. [39]	2023	Case series	5	HBOT was suggested as an alternative non-invasive method for long COVID treatment characterized by oxy-inflammation.
Pan et al. [40]	2023	-	-	HBOT sustains the improvement of symptom severity, reducing duration, and enhancing patients’ quality of life.
Jermakow et al. [41]	2025	Case series	30	HBOT may help as an adjunctive treatment for PASC patients by increasing oxygen saturation and tuning the immune response.

## Data Availability

No new data were created or analyzed in this study.
